# Risk Factors for Neck Nodal Metastasis in Papillary Thyroid Cancer With BRAF V600E Mutation

**DOI:** 10.3389/fendo.2022.884428

**Published:** 2022-06-16

**Authors:** Ying Han, Ling Hou, Bowen Zhao, Li Gao, Shiyan Li

**Affiliations:** ^1^ Department of Ultrasound, Sir Run Run Shaw Hospital, School of Medicine, Zhejiang University, Hangzhou, China; ^2^ National Clinical Research Center for Child Health, The Children’s Hospital, School of Medicine, Zhejiang University, Hangzhou, China; ^3^ Department of Head and Neck Surgery, Sir Run Run Shaw Hospital, School of Medicine, Zhejiang University, Hangzhou, China

**Keywords:** BRAF V600E, thyroid carcinomas, lymph node, metastasis, multivariate analysis, risk factors

## Abstract

**Background:**

The BRAF V600E mutation is the most common genetic variant in papillary thyroid cancer (PTC), but the relationship between the BRAF V600E mutation in PTC and cervical lymph node metastasis (LNM) remains controversial.

**Objective:**

To estimate risk factors for neck nodal metastasis in PTC with BRAF V600E mutation.

**Patients:**

A total of 292 patients diagnosed with BRAF V600E mutation related PTC were admitted.

**Design:**

In this retrospective study, data from 292 patients, including clinical, molecular, and ultrasonic characteristics, were analyzed. Univariate and multivariate logistic regression analyses were applied to identify risk factors for LNM in PTC with the *BRAF* V600E mutation.

**Results:**

In the univariate analysis of all PTC patients with the BRAF V600E mutation, the LNM was found to be significantly associated with age (*P* = 0.010), size (*P* = 0.000), bilaterality (*P* = 0.000), multifocality (*P* = 0.002), LNM in ultrasound (US) (*P* = 0.000), and capsular invasion (*P* = 0.010). In ultrasonic image characteristics, margin (*P* = 0.036), shape (*P* = 0.046), and microcalcification (*P* = 0.002) were significantly associated with LNM. In multivariate analysis, LNM in PTCs with BRAF V600E mutation was significantly associated with age ≤ 45 years (OR = 1.869, *P* = 0.020, 95% CI = 1.106 - 3.158), size ≥ 1cm (OR = 3.131, *P* = 0.001, 95% CI = 1.578 - 6.212), LNM in US (OR = 6.962, *P* = 0.000, 95% CI = 2.924 - 16.572), bilaterality (OR = 1.626, *P* = 0.007, 95% CI = 1.142 - 2.314), ill-defined margins in US (OR = 1.980, *P* = 0.033, 95% CI = 1.057 - 3.709), and microcalcification in US (OR = 2.786, *P* = 0.002, 95% CI = 1.464 - 5.303).

**Conclusion:**

This study revealed that several significant risk factors for LNM in PTCs with the BRAF V600E mutation included: age ≤ 45 years, size ≥ 1cm, LNM in US, bilaterality, ill-defined margins in US, and microcalcification in US.

## Introduction

Over the last decades, thyroid cancer has been one of the fastest growing and most prevalent endocrine malignancies worldwide (1, 2). It is 2.9 times more common in women than in men, possibly due to reproductive and hormonal factors (2). Papillary thyroid carcinoma (PTC) accounts for approximately 85% of thyroid cancers, as the most common differentiated thyroid carcinomas (DTC) with a favorable prognosis (3). In recent years, molecular markers have gained extensive attention to improving risk stratification of PTC (4).

In PTCs, the most common mutations are BRAF mutations, which were reported in 40 - 90% of cases from various geographical areas (5–7). BRAF is the main subtype of RAF kinase that may contribute to cell proliferation, growth, division and plays a key role in tumorigenesis (7). The mutation of BRAF V600E could trigger tumorigenesis through constitutively activating the MAPK pathway (8, 9). However, the function of BRAF V600E as a biomarker in driving aggressiveness in PTC continues to be debatable because of the limited large cohort. To date, the relationship between the BRAF V600E mutation in PTC and cervical lymph node metastasis (LNM), remains disputable. The majority of studies claim that the BRAF V600E mutation was associated with poor clinicopathologic outcomes in patients with PTC, including neck nodal metastasis (8, 10, 11). By contrast, some reports suggest that the BRAF V600E variant had no significant association with neck nodal metastasis (7, 12, 13). Therefore, it is still controversial whether the BRAF V600E mutation is associated with cervical LNM. We suspect that this may be due to some selection bias in the tumors included in previous studies, such as large tumor size, multifocality, and bilateral tumors.

In this study, we retrospectively analyzed the clinical, molecular and ultrasonic characteristics in PTC with BARF V600E mutation to find out the influencing factors of cervical LNM.

## Patients and Methods

### Study Population and Design

The clinical records of 487 patients who underwent genotyping for 77 PTC-associated variants and partial or total thyroidectomy in Sir Run Run Shaw Hospital from January 2018 to October 2021 were collected. We obtained written informed consents for all patients, their relatives for FNA, and the collection of DNA samples from FNA or surgery for next generation sequencing. All the procedures described in this study were in accordance with institutional ethical standards. We included patients according to the following criteria: ① PTC confirmed by surgical pathology, ② PTC with BRAF V600E mutation, ③ presence or absence of LNM was pathologically confirmed, ④ complete clinical, molecular, and imaging data, ⑤ age ≥18 years old for all 487 patients. A total of 195 patients were excluded according to the following criteria: ① the final pathological findings were benign or non-PTC malignancy, ② patients were without BRAF V600E mutation, ③ lack of complete clinical, molecular, or imaging data, ④ a history of previous thyroid surgery, ⑤ other extra-thyroid malignancies, and ⑥ age <18 years old. Finally, 292 patients were enrolled in this research.

In this study, the patient’s age, sex, size of the tumor (maximum diameter in US), LNM (central or lateral cervical) in pathology, LNM in US, occurrence of capsular invasion, presence of Hashimoto’s thyroiditis (HT), focality (unifocal or multifocal), lobe of tumor (left or right), combination of BRAF V600E and other gene variants, ultrasonic features of tumor (location, margin, shape, internal content, echogenicity, calcification) were analyzed. We selected unifocal tumors for ultrasonic characteristic analysis.

### Ultrasonic Characteristic Analysis

US examination was performed by sonographers specializing in head and neck imaging, especially in thyroid, who have 10–20 years of experience. The sonographers who performed the US examination prospectively recorded the US imaging features of the thyroid tumors and central or lateral cervical lymph node status. The ultrasonic image information of thyroid tumors we collected included size, location, composition, margin, shape, echogenicity, and echogenic foci. Lymph nodes with suspicious metastasis by US had the following characteristics: cystic aspect, microcalcification, peripheral vascularity, hyperechogenicity, round shape, and loss of hilum (14). If more than one nodule with suspicion of malignancy was found in thyroid gland, the maximum diameter of lesions was recorded and included in the data analysis.

### Molecular Analysis

DNA mutation analysis was performed using customized next generation sequencing on an Illumina (San Diego, CA, USA) MiSeq targeting 77 genes (including BRAF, RAS, TP53, TERT, NOTCH1, TSHR, ATM, APC, CCDC6/RET, etc.) at the Molecular Pathology Diagnostic Center in Sir Run Run Shaw Hospital, Zhejiang University School of Medicine. All identified mutations were validated by Sanger sequencing and matched-normal gDNA. All samples were obtained from FNA or surgical specimens. We considered the tumor as mutated for a particular gene if, at least, one of the samples harbored a mutation when multiple samples from different areas were tested.

### Statistical Analysis

Data were analyzed using SPSS 25.0 software (IBM). Measurement data were presented as mean ± SD. or median and range. Categorical data were summarized with frequencies and percentages. Pearson c^2^ test and Fisher’s exact test were used to calculate the bivariate analysis of the relationship between LNM and clinical, molecular, or ultrasonic features in PTCs with the BRAF V600E mutation. A multivariate analysis was performed using binary logistic regression analysis for variables, which were significant in the bivariate analysis. The odds ratio (OR) value and 95% CI were reported. *P* values less than 0.05 were considered statistically significant.

## Results

### Patient Characteristics

In our study, 292 patients diagnosed with the BRAF V600E mutation related PTC were included. The average age ( ± SD) was 45.6 ± 11.8 years (range, 23–77 years), and 77.4% of the patients were female (226 women and 66 men). In all PTCs, cervical LNM (100 central, 4 lateral cervical, 43 central, and lateral cervical) occurred in 50.3% of patients. A total of 175 patients with single-focal tumors were included for the ultrasonic characteristic analysis. **Tables 1**–**3** show the clinical, ultrasonic, and molecular factors of the patients.

**Table 1:** Association between LNM and clinical, molecular characteristics of PTCs with the BRAF V600E mutation.

Variables	LNM (%)		X^2^	*P* value
	-	+		
Sex				
Female	118 (52.2)	108 (47.8)	2.611	0.106
Male	27 (40.9)	39 (59.1)		
Age (years)				
≤45	64 (42.4)	87 (57.6)	6.618	0.010
>45	81 (57.4)	60 (42.6)		
Size (cm)				
<1	129 (58.1)	93 (41.9)	26.454	0.000
≥1	16 (22.9)	54 (77.1)		
Focality				
Unifocal	100 (57.1)	75 (42.9)	9.789	0.002
Multifocal	45 (38.5)	72 (61.5)		
Lobe				
Left^a^	58 (56.3)	45 (43.7)	17.094	0.000
Right^a^	69 (56.1)	54 (43.9)		
Bilaterality^b^	18 (27.3)	48 (72.7)		
LNM in US				
+	7 (12.1)	51 (87.9)	40.906	0.000
–	138 (59.0)	96 (41.0)		
Capsular invasion				
+	7 (25.9)	20 (74.1)	6.702	0.010
–	138 (52.1)	127 (47.9)		
HT				
+	41 (56.2)	32 (43.8)	1.648	0.199
–	104 (47.5)	115 (52.5)		
Other variants				
+	45 (42.5)	61 (57.5)	3.455	0.063
–	100 (53.8)	86 (46.2)		

LNM, lymph node metastasis; PTC, papillary thyroid cancer; US, ultrasound; HT, Hashimoto’s thyroiditis; ^a^, pairwise comparisons were not statistically significant; ^b^, pairwise comparisons were statistically significant (P < 0.05).

**Table 2:** Association between LNM and US characteristics of PTCs with the BRAF V600E mutation.

Variables	LNM (%)		X^2^	*P* value
	-	+		
Location				
Upper third	30 (53.6)	26 (46.4)	2.450	0.484
Middle third	22 (56.4)	17 (43.6)		
Lower third	46 (62.2)	28 (37.8)		
Isthmus	2 (33.3)	4 (66.7)		
Margin				
Smooth	56 (65.1)	30 (34.9)	4.390	0.036
Ill-defined	44 (49.4)	45 (50.6)		
Shape				
Oval	27 (46.6)	31 (53.4)	3.974	0.046
Taller-than-wide	73 (62.4)	44 (37.6)		
Internal content				
Solid	99 (57.2)	74 (42.8)	0.042	0.837
Cystic and solid	1 (50.0)	1 (50.0)		
Echogenicity				
Hypoechogenicity	100 (57.8)	73 (42.2)	2.697	0.101
Iso/Hyperechogenicity	0 (0.0)	2 (100.0)		
Microcalcification				
+	47 (47.0)	53 (53.0)	9.802	0.002
–	53 (70.7)	22 (29.3)		

LNM, lymph node metastasis; PTC, papillary thyroid cancer; US, ultrasound; microcalcification, calcification < 0.2 cm in diameter.

**Table 3:** LNM in PTC patients with combined mutations in BRAF V600E and other genes.

Other variants	Total	LNM (%)	Other variants	Total	LNM (%)
NOTCH1	20	12 (60.0)	IDH1	6	2 (33.3)
RET	16	10 (62.5)	AXIN1	5	3 (60.0)
RAS	13	7 (53.8)	TSC2	5	3 (60.0)
KMT2C	12	5 (41.7)	TERT	4	4 (100.0)
ZFHX3	12	8 (66.7)	TSHR	4	1 (25.0)
NCOR2	10	4 (40.0)	SPOP	4	1 (25.0)
TP53	7	5 (71.4)	CCDC6/RET	4	2 (50.0)
APC	3	0 (0.0)	DICER1	2	0 (0.0)
ETV6/NTRK3	2	2 (100.0)	PTEN	2	2 (100.0)

LNM, lymph node metastasis; PTC, papillary thyroid cancer.

### Association of LNM and Clinical, Molecular, and Ultrasonic Characteristics of PTCs with BRAF V600E Mutation

In the univariate analysis of clinical and molecular features in all PTCs with BRAF V600E mutation (**Table 1**), the LNM was found to be significantly associated with age (*P* = 0.010), size (*P* = 0.000), lobe (*P* = 0.000), focality (*P* = 0.003), LNM on US (*P* = 0.000), and capsular invasion (*P* = 0.010). However, no significant association was found between LNM and other clinical characteristics, such as sex (*P* = 0.106), HT (*P* = 0.199), and combination of BRAF V600E with other variants (*P* = 0.063).

In ultrasonic image characteristics of all unifocal PTCs with BRAF V600E mutation (**Table 2**), location (*P* = 0.484), internal content (*P* = 0.837), and echogenicity (*P* = 0.101) were not statistically significant variables associated with LNM. However, margin (*P* = 0.036), shape (*P* = 0.046), and microcalcification (*P* = 0.002) were significantly associated with LNM.

The combination of BRAF V600E with other mutations is shown in **Table 3**. A total of 18 other gene mutations and fusions were identified, including mutations carried by other thyroid tumors from the same patient, in addition to the BRAF V600E mutated PTC. The frequency of LNM was 100.0% in patients with TERT promoter and PTEN mutation and 71.4% in patients with TP53 mutation.

### Multivariate Logistic Regression Analysis

In multivariate analysis, LNM in PTCs with BRAF V600E mutation was significantly associated with age ≤ 45 years (OR = 1.869, *P* = 0.020, 95% CI = 1.106 - 3.158), size ≥ 1cm (OR = 3.131, *P* = 0.001, 95% CI =1.578 - 6.212), LNM on US (OR = 6.962, *P* = 0.000, 95% CI = 2.924 - 16.572), bilaterality (OR = 1.626, *P* = 0.007, 95% CI = 1.142 - 2.314), ill-defined margins (OR = 1.980, *P* = 0.033, 95% CI = 1.057 - 3.709), and microcalcification (OR = 2.786, *P* = 0.002, 95% CI = 1.464 - 5.303), as shown in **Table 4**.

**Table 4:** Multivariate logistic regression analyses: association between LNM and clinical/ultrasonic characteristics of PTCs with the BRAF V600E mutation.

Characteristics	OR	*P* value	95% CI for OR	
			Lower	Upper
Age ≤ 45 years	1.869	0.020	1.106	3.158
Size ≥ 1 cm	3.131	0.001	1.578	6.212
LNM in US	6.962	0.000	2.924	16.572
Bilaterality	1.626	0.007	1.142	2.314
Ill-defined margin	1.980	0.033	1.057	3.709
Microcalcification	2.786	0.002	1.464	5.303

LNM, lymph node metastasis; PTC, papillary thyroid cancer; US, ultrasound; microcalcification, calcification < 0.2 cm in diameter.

## Discussion

As one of the most common molecular markers, the BRAF V600E mutation has received wide attention. Many studies have considered that BRAF V600E mutation as independently related to known prognostic factors such as LNM (8, 10, 11). However, some studies take the opposite view (7, 12, 13). This study sought to find the influence factors associated with LNM in PTC with the BRAF V600E mutation and clarify the probable reasons for the disagreement over whether BRAF V600E mutation is associated with LNM. Celik et al. (15) suggested that this difference among the previous studies might be due to different treatment protocols used in different institutions, since LNM is highly detectable when prophylactic lymph node dissection is performed. Whereas, we hypothesized that this difference might be related to selection bias caused by some factors. In this study, we conducted univariate and multivariate logistic regression analyses of the clinical, molecular, and ultrasonic characteristics of PTCs with the BRAF V600E mutation to identify the relevant influencing factors.

Frequency of LNM in PTCs with the BRAF V600E mutation were reported to range from 48–70% from different institutions (7, 12, 15–18). In our research, the incidence of LNM was 50.3%. This is consistent with previous reports. Based on univariate analysis of 292 patients (**Table 1**), sex and HT coexisting with other variants, showed no significant association with LNM in BRAF-related PTCs. In patients with BRAF V600E mutated PTC, LNM was less frequent in subjects with HT than those without HT (43.8% vs. 52.5%), but there was no statistically significant difference. This is similar to the results of Marotta ‘s report (19). Other research (20) showed that HT was related to less lymph node metastasis and extrathyroidal extension in PTCs with the BRAF V600E mutation and may play a protective factor in PTCs by directly or indirectly inhibiting the expression of the BRAF V600E mutation and reducing the presence of the aggressive factors in PTCs with BRAF V600E mutation. Further experiments are needed to clarify whether there is a connection between BRAF V600E and HT in the occurrence and progression of thyroid cancer. In molecular terms, we did not find a significant association between LNM and combined mutations. As shown in **Table 3**, the frequency of LNM in PTCs with concomitant BRAF and TERT promoter mutations was up to 100%. Several studies have supported that the TERT promoter mutated PTC has poor prognosis and clinical staging and the synergistic effects of concomitant BRAF and TERT promoter mutations may exist (9, 21, 22). The rate of LNM was 71.4% in patients with coexistent mutations of BRAF V600E and TP53. This confirms the previous view that TP53 mutations convey significant adverse prognosis and are associated with tumor progression (23, 24).

In addition, 2 patients with combined PTEN mutation both showed cervical LNM in our research, which may mean that the PTEN variant is also associated with tumor growth, aggressiveness, and poor prognosis (25, 26).

In univariate analysis, LNM were significantly associated with age (≤ 45 years), size (≥ 1cm), bilaterality, multifocality, LNM on US, capsular invasion, ultrasonic image features (margin, shape, microcalcification) in BRAF mutated PTCs. Multifocality, capsular invasion, shape showed insignificant after multivariate adjustment. In the multivariate logistic analysis, our results indicated that patients >45 years old had less frequent LNM. Mao’s report (27) defined several significant risk factors of cervical LNM in PTC patients: age (<45 years), gender (male), multifocality, tumor size (>1 cm), tumor location (1/3 upper), capsular invasion by the systematic review, and meta-analysis and showed bilateral tumors were unrelated to LNM in patients with PTC, regardless of the BRAF V600E mutation. The BRAF V600E mutation, gender, multifocality, tumor location, and capsular invasion had no significance for LNM but bilateral tumors are associated with LNM in BRAF V600E-related PTCs. Consistent with our findings, Shi’s result (28) shows that incidence of LNM in PTC patients was significantly higher when the tumor diameter was >1cm, while when the tumor diameter was ≤ 1cm, the BRAF V600E mutation status had no effect on LNM. With regard to the conventional US features, Xu et al. (29) reported that solid component, marked hypoechogenicity or hypoechogenicity, microcalcification, ill-defined margins, and taller than wide shape were all not significant in positive BRAF V600E mutation patients with central LNM or lateral LNM. On the contrary, in the present study, US features including ill-defined margins and microcalcification were associated with LNM in positive BRAF V600E mutation PTCs. Some important US predictors might be under debate in certain circumstances. This may be due to the selection bias that occurred in this retrospective unicentral research with limited number of cases. Therefore, further research needs to be performed from multiple centers with a large sample of patients in the future.

## Conclusion

This study revealed that several significant risk factors for LNM in PTCs with BRAF V600E mutation included: age ≤ 45 years, size ≥ 1cm, LNM in US, bilaterality, ill-defined margins in US, microcalcification in US.

## Data Availability Statement

The original contributions presented in the study are included in the article/**Supplementary Material**. Further inquiries can be directed to the corresponding author.

## Ethics Statement

The studies involving human participants were reviewed and approved by Ethics Committee of Sir Run Run Shaw Hospital, Zhejiang University School of Medicine. The patients/participants provided their written informed consent to participate in this study. Written informed consent was obtained from the individual(s) for the publication of any potentially identifiable images or data included in this article.

## Author Contributions

YH was responsible for conceptualization, study design, data collection and analysis, writing of the manuscript. LH and YH have contributed equally to this work. BZ and LG contributed to study design, writing-reviewing. SL contributed to conceptualization, study design, funding acquisition, writing-reviewing and editing. All authors approved the final version of the manuscript.

## Funding

This work was supported by the Natural Science Foundation of Zhejiang Province (LY20H180005).

## Conflict of Interest

The authors declare that the research was conducted in the absence of any commercial or financial relationships that could be construed as a potential conflict of interest.

## Publisher’s Note

All claims expressed in this article are solely those of the authors and do not necessarily represent those of their affiliated organizations, or those of the publisher, the editors and the reviewers. Any product that may be evaluated in this article, or claim that may be made by its manufacturer, is not guaranteed or endorsed by the publisher.
